# Complete mitochondrial genome and draft chloroplastic genome of *Haslea ostrearia* (Simonsen 1974)

**DOI:** 10.1080/23802359.2023.2268747

**Published:** 2023-10-13

**Authors:** Aurelie Peticca, Mostefa Fodil, Helene Gateau, Jean-Luc Mouget, Francois Sabot, Benoit Chenais, Nathalie Casse

**Affiliations:** a BiOSSE (Biology of Organisms: Stress, Health, Environment), UFR Sciences et Techniques, Le Mans Universite, Le Mans, France; b DIADE, University of Montpellier, CIRAD, Montferrier-sur-Lez, France

**Keywords:** *Haslea ostearia*, diatom, blue microalgae, chloroplast, mitochondrion

## Abstract

The first completed, circular mitochondrial genome and the first draft, linear chloroplastic genome of the blue diatom *Haslea ostrearia* (Simonsen 1974, *Naviculaceae, Bacillariophyceae*) were assembled from Illumina and PacBio sequencing. The mitochondrial genome was composed of 38,696 bases and contained 64 genes, including 31 protein-coding genes (CDS), 2 ribosomal RNA (rRNA) genes and 23 transfer RNA (tRNA) genes. For the chloroplast, the genome was composed of 130,200 bases with 169 genes (131 CDS, 6 rRNA genes, 31 tRNA genes, and 1 transfer messenger RNA gene). Phylogenetic trees, using the maximum-likehood method and partial genes currently available for *Haslea ostrearia* and other diatom species, suggested the proximity of all the *Haslea ostrearia* strains/isolates and the possibility of using these genomes as future references.

## Introduction

*Haslea* (*H.*) *ostrearia* is a blue microalga from the *Naviculaceae* family, which lives freely in benthic marine environments or as an epiphyte on brown macroalgae (Simonsen 1974, Figure 1). The blue color comes from a pigment called marenine, that *H. ostrearia* produces and accumulates at cell apices (Gastineau et al. 2014a). This specific pigment is responsible for the greening of oyster gills in farming ponds in Western France. Furthermore, it has been shown that marenine could display antibacterial, antiviral and antifungal effects (Gastineau et al. 2014b). However, *H. ostrearia* is still very much unknown, especially at the genetic level. One of the reasons for this lack of knowledge is that this microalga needs many bacteria to survive (Lepinay et al. 2016, 2018). Their excessive presence confuses the sequencing data. However, the complete DNA of one of its representatives from the North Atlantic Ocean has been sequenced, and allowed the reconstruction of its mitochondrial and chloroplastic genomes. This study clears the way for future research about *H. ostrearia* and will help characterize the largely unknown genetics of this species. In particular, it will serve as a future basis for the taxonomic classification of this species, but also as a potential marker for finding the presence of *H. ostrearia* in metagenomic sequencing.

**Figure 1.**
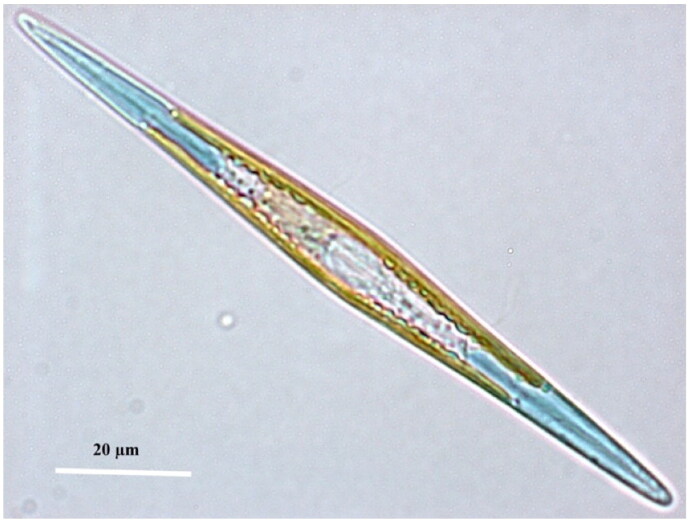
‘Living cell of *Haslea ostrearia* observed in light microscopy’ from Gabed et al. (2022).

## Material

The isolate used in this study was collected in 2018 from an oyster pond in Bouin, France (latitude 46.953444 and longitude −2.046139) and deposited in the Nantes Culture Collection (curator Vona Meleder, vona.meleder@univ-nantes.fr; Nantes, France) under the name NCC 532.

## Methods

During 21 days, the culture was grown in enriched artificial sea water (*Instant Ocean*, Aquarium systems O; Harrison et al. 1980 modified by De Brouwer et al. 2002) at 14 °C under 300 μm photons/m^2^/s with a 14h/10h light/dark cycle. On the 20th day of growth, a 1:100 dose of Sigma’s antibiotic antimycotic solution (Sigma-Aldrich, Saint-Quentin Fallavier, France; catalogue#A5955) was added to the culture mix. After 24h, the biomass was collected through filtration (Whatman™ Binder-Free Glass Microfiber Filters, Grade GF/C, pore size of 1.2 μm) and the whole DNA was extracted using the method of Puppo et al. (2017). Extracted DNA was sequenced using the PacBio continuous long reads (PB CLR SEQUEL2) and Illumina MiSeq platforms (TrueSeqv3, 150pe; Genotoul, Toulouse, France), and 85 Gb and 5.241 Gb total read lengths were generated, respectively. Illumina reads were filtered to remove low-quality reads (<Q30), short reads (<75b) and adapter sequences were searched and trimmed by trimmomatic v0.39 (Bolger et al. 2014). The *de novo* genome assembly was performed using Flye v2.9 (Kolmogorov et al. 2020) and the long PB CLR reads with the following options: -g 100 m –meta. Polishing was performed with filtered Illumina reads through three loops of racon v1.4.20 (Vaser et al. 2017) and bwa-mem v2 2.2.1 (Vasimuddin et al. 2019). As the sequencing data also contained bacteria, due to the characteristics of this microalgae which did not seem to be able to survive without them (Lepinay et al. 2016, 2018). This is also the reason for the decision to sequence the data in long and short reads, as it would have been difficult to obtain a quality assembly with only one of the latter (Chen et al. 2022). The mitochondrial and chloroplast genomes were identified using minimap2 v2.18 (Li 2018) by aligning the metagenome obtained here against the *H. nusantara* mitochondrion (MH681882 accession number, NCBI database, https://www.ncbi.nlm.nih.gov/) and the *Phaeodactylum tricornutum* chloroplast (NC_008588, NCBI database). The sequences aligned with a percent identity superior to 80 were retained (%ID). The annotation and gene prediction were performed by Prokka v1.14.6 (Seemann 2014). Since the mitochondrial genome is circular, samtools faidx v1.14 (Danecek et al. 2021) was used to relocate the annotated origin of H-strand replication (OH) as the starting gene (position +1). Attempts to make the chloroplast circular using Circlator v1.5.5 (Hunt et al. 2015) were unsuccessful, as the sequences may be too dense at some point. Downsampling seems to be the solution to prevent an assembly from failing due to information overload (Mirebrahim et al. 2015), but unfortunately with the over-representation of bacteria this method cannot be applied. The Supplementary Figure 1 was made according to the ‘Generating Sequencing Depth and Coverage Map for Organelle Genomes’ in protocol.io (https://www.protocols.io/view/generating-sequencing-depth-and-coverage-map-for-o-4r3l27jkxg1y/v1) using the assembled genome, the PacBio reads, minimap2 2.18 (Li 2018) and Samtools 1.14 (Danecek et al. 2021). Genome maps were generated with Artemis v18.2.0 (Rutherford et al. 2000).

Phylogenetic trees were created with NGPhylogeny.fr pipeline (trimAl and PhyML + SMS, Lemoine et al. 2019) with the partial *COX1* gene for the mitochondrial and the partial *rbcL* gene chloroplast genomes, as these are the only data available for this species. The mitochondrial dataset grouped sequences from different diatom taxa: 5 *H. ostrearia* strains, 4 other *Haslea* species, 5 *Navicula* species and 2 external species from the *Eunotia* family. The chloroplast dataset al.so included different diatom sequences: 3 *H. ostrearia* strains, 5 other *Haslea* species, 8 *Navicula* species and 2 external species from the *Eunotia* family. All sequences were downloaded from NCBI database (https://www.ncbi.nlm.nih.gov/), and their access number available on the phylogenetic trees.

## Results

**Figure 2.**
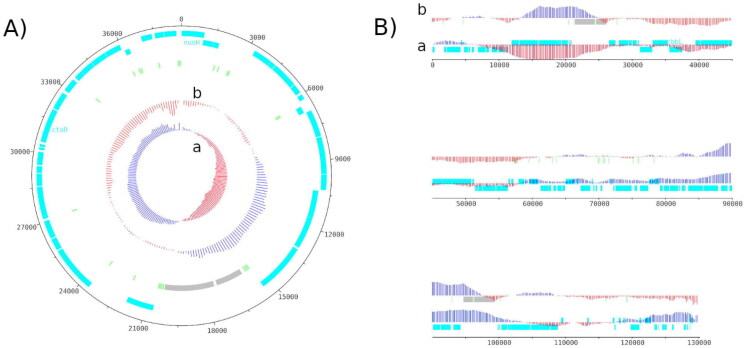
Genome map of the mitochondrial genome (A) and the chloroplastic genome (B) of *H. ostrearia.* The red and blue colors in GC shiew (a) and GC plot (b) show if the value is below or above average. Protein-coding genes are shown in light blue, transfer RNA genes in light green and ribosomal RNA in light grey. The chloroplast genome is shown as a linear genome because it is not complete. The genome map was made using Artemis v18.2.0.

The complete circular mitochondrial genome was 38,696 bases long with a GC content (%GC) of 28.66% (36.07% A, 35.26% T, 14.76% C, 13.91% G), with a sequencing depth of 1,325X. The 64 annotated genes were composed of 39 protein-coding genes (CDS), 2 ribosomal RNA (rRNA) genes and 23 transfer RNA (tRNA) genes (Figure 2A). The *H. ostrearia COX1* gene, previously partially sequenced (Gastineau et al. 2013), was found complete in this study and annotated as *ctaD* by Prokka. The draft chloroplast genome was 130,200 bases long with 31.04%GC (34.19 A, 34.76% T, 15.31% C, 15.73% G) and with a sequencing depth of 2,371X. One hundred and thirty one CDS, 6 rRNA genes, 31 tRNA genes and 1 tmRNA gene were identified within this genome (Figure 2B).

**Figure 3.**
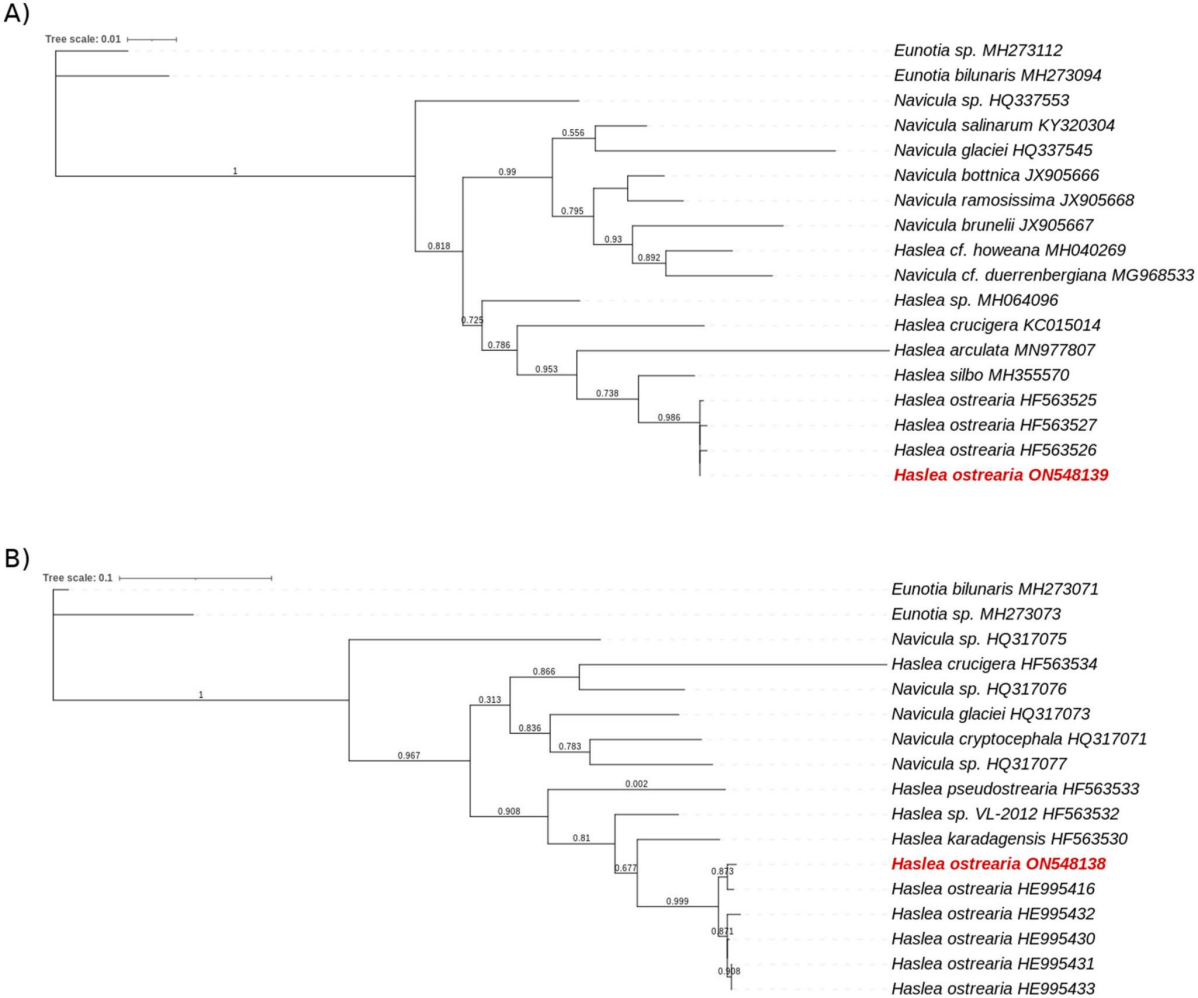
Maximum likelihood phylogenetic trees inferred from *COX1* and *rbcL* genes from diatoms genus. The phylogenetic trees was performed with, respectevily, *Haslea ostrearia* (in red) and 16 to 18 other diatom chloroplastic gene *rbcL* (A) and mictochondrial gene *COX1* (B). Numbers near the nodes indicate bootstrap support values. The accession number associated with each gene is listed next to the species name. *Eunotia* species were used as external species. NGPhylogeny.fr pipeline was used to generate these phylogenetic trees.

The phylogenetic tree obtained for the *rbcL* gene from diatoms chloroplastic genomes showed a clear separation between *Navicula* and *Haslea* species, with a bootstrap value of 0.818 (Figure 3A), the only exception being the *rbcL* gene of *H. howeana* found among *Navicula*. Looking in details, the *H. ostrearia* genes were clustered in the same part of the tree with very small branches. They were also separated from the other diatoms by a branch with a bootstrap value of 0.986. The *Eunotia* genes, taken as external species, were well observed on the external branches of the tree. The same observations were made for the mitochondrial gene *COX1* (Figure 3B). *H. crucigera* was the only *Haslea* species found the *Navicula*. The *COX1* genes from an *H. ostrearia* were all aggregated with small branches and were separated from the others by a bootstrap value of 0.999. Due to a lack of information about genes in the genus *Haslea,* only the partial *COX1* and the *rbcL* genes were tested.

## Discussion and conclusion

Given the lack of information for the genus *Haslea*, the genomes reconstructed here could only be compared directly with those of one other species to check their completeness. Both appear to be close to the chloroplast and mitochondria of *H. nusantara.* The mitochondrion of the latter was 36,288 bases long for a 29.24%GC, and the chloroplast was 120,448 bases long for a 31.10%GC (Prasetiya et al. 2019), *i.e.* 2 kb and 10 kb more than those of *H. ostrearia.* The 64 annotated genes for the *H. ostrearia* mitochondrion were slightly more numerous than *H. nusantara* ones with 3 additional CDS and one less tRNA (Prasetiya et al. 2019). Among the genes found here is *COX1*, which has been identified as being highly conserved in the mitochondria of the *Haslea* (Gastineau et al. 2013). The mitochondria of *H. ostrearia* appeared to be colinear with those of *H. nusantara*, the same synteny was observed in their genomes in the form of three distinct blocks. The same comment could be made for the chloroplast, **t**he structure and the genes were very similar to the *H. nusantara* ones. The two chloroplast genomes closely resembled each other, sharing close to 90% sequence identity. The only exceptions were a missing part of the genome corresponding to ∼10 kb of the *H. nusantara* chloroplast (positions around 90,000-104,000) and the sequence inversions observed between *H. ostrearia* and *H. nusantara* for the first 70,000 bases of the latter. It was also very similar to the general features of diatom chloroplast genomes from Prasetiya et al. (2019) study. This amplified the idea that the genomes are complete for the mitochondria and almost complete for the chloroplast, even if the latter has not been circularized. In addition, the already known *H. ostrearia rbcL* gene, annotated as *cbbL*, and the *psbC* partial gene were also found completed in this study (Gastineau et al. 2013). Phylogenetic trees of both mitochondrial and chloroplastic sequences supported the hypothesis that *H. ostrearia* strains used here were very close to each other and had a different evolutionary history from other diatoms or *Haslea* species (Figure 3). High read coverage was also observed for each genome, respectively 1389X and 2321X for the mitochondrial and the chloroplastic sequences (Supplementary Figure 1). In the case of the latter, there is a short drop of up to 6X in coverage, but the average observed over the 4,000 bases it represents is ∼200X which is above the 30X traditionally desired for a *de novo* assembly.

Even if the chloroplast genome was not complete, it seemed that only a few bases were missing. Its size exceeded that of the plastid from the close species *H. nusantara,* but with a highly similar composition (%GC and genes number). Since all *H. ostrearia* strains, for which DNA sequences are available, presented a close proximity to each other, the mitochondrial and chloroplast genomes presented here could be used as a reference for this species.

## Supplementary Material

Supplemental Material

## Data Availability

The genome sequence data that support the findings of this study are openly available in GenBank of NCBI at https://www.ncbi.nlm.nih.gov/ under the accession no. ON548138 and ON548139. The associated BioProject, SRA and Bio-Sample numbers are PRJNA843895, SRR19450090/SRR19450089, and SAMN28772203 respectively.
