# Supplementary material for: Complete mitochondrial genome and draft chloroplastic genome of Haslea ostrearia (Simonsen 1974)
Source: Mitochondrial DNA B Resour. 2023 Oct 13;8(10):1092–6. doi: 10.1080/23802359.2023.2268747 (PMC10578087; doi:10.1080/23802359.2023.2268747)
Supplement: Supplemental Material [file TMDN_A_2268747_SM3165.pdf]

Supplementary material

A)

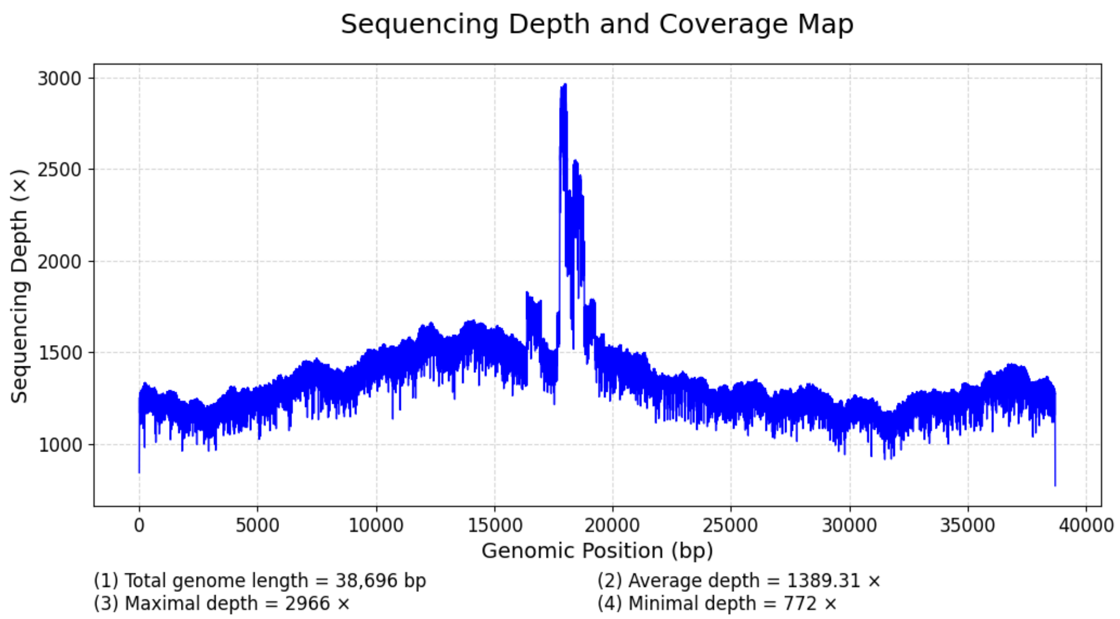

B)

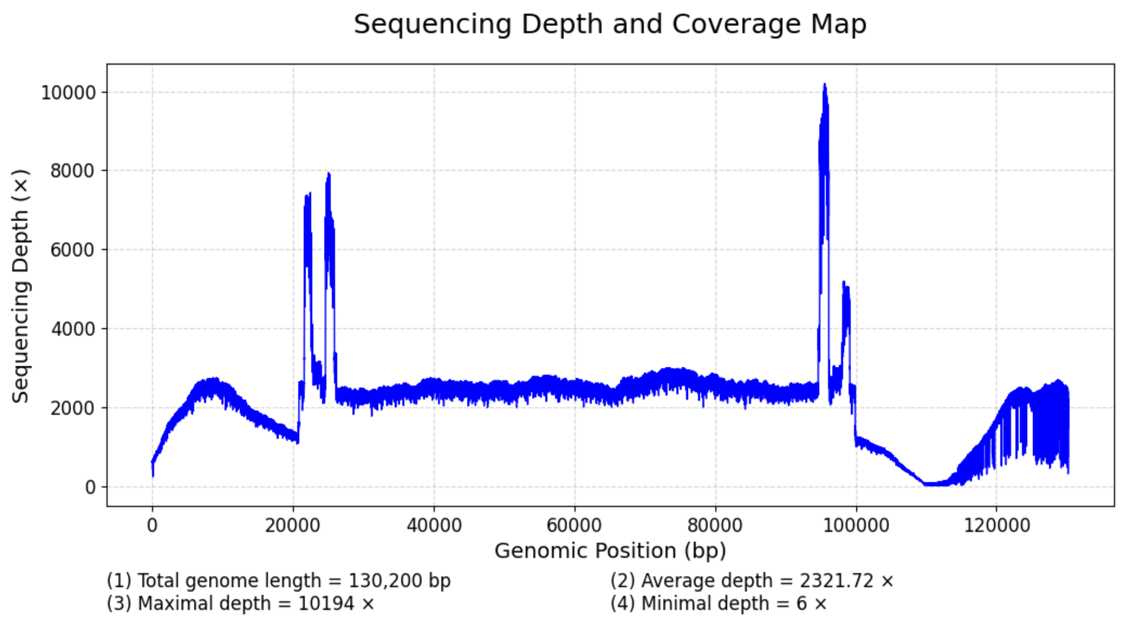

<u>Supplementary Figure 1</u>: Pacbio read coverage depth map of the mitochondrial genome (A) and the chloroplastic genome (B) of *H. ostrearia*
